# Exosomal Metabolic Signatures Are Associated with Differential Response to Neoadjuvant Chemotherapy in Patients with Breast Cancer

**DOI:** 10.3390/ijms23105324

**Published:** 2022-05-10

**Authors:** Shriya Joshi, Chakravarthy Garlapati, Shristi Bhattarai, Yixin Su, Leslimar Rios-Colon, Gagan Deep, Mylin A. Torres, Ritu Aneja

**Affiliations:** 1 Department of Biology, Georgia State University, Atlanta, GA 30303, USA; sjoshi8@student.gsu.edu (S.J.); cgarlapati1@student.gsu.edu (C.G.); shristi.bhattarai@gmail.com (S.B.); 2 Department of Cancer Biology, Wake Forest School of Medicine, Winston-Salem, NC 27157, USA; ysu@wakehealth.edu (Y.S.); lrioscol@wakehealth.edu (L.R.-C.); gdeep@wakehealth.edu (G.D.); 3 Julius L. Chambers Biomedical Biotechnology Research Institute, North Carolina Central University, Durham, NC 27707, USA; 4 Department of Radiation Oncology, Emory University School of Medicine, Atlanta, GA 30322, USA; matorre@emory.edu; 5 Department of Clinical and Diagnostic Sciences, School of Health Professions, University of Alabama at Birmingham, Birmingham, AL 35294, USA

**Keywords:** breast cancer, neoadjuvant chemotherapy, pathologic complete response, residual disease, exosomes, metabolomics

## Abstract

Neoadjuvant chemotherapy (NAC) is commonly used in breast cancer (BC) patients to increase eligibility for breast-conserving surgery. Only 30% of patients with BC show pathologic complete response (pCR) after NAC, and residual disease (RD) is associated with poor long-term prognosis. A critical barrier to improving NAC outcomes in patients with BC is the limited understanding of the mechanisms underlying differential treatment outcomes. In this study, we evaluated the ability of exosomal metabolic profiles to predict NAC response in patients with BC. Exosomes isolated from the plasma of patients after NAC were used for metabolomic analyses to identify exosomal metabolic signatures associated with the NAC response. Among the 16 BC patients who received NAC, eight had a pCR, and eight had RD. Patients with RD had 2.52-fold higher exosome concentration in their plasma than those with pCR and showed significant enrichment of various metabolic pathways, including citrate cycle, urea cycle, porphyrin metabolism, glycolysis, and gluconeogenesis. Additionally, the relative exosomal levels of succinate and lactate were significantly higher in patients with RD than in those with pCR. These data suggest that plasma exosomal metabolic signatures could be associated with differential NAC outcomes in BC patients and provide insight into the metabolic determinants of NAC response in patients with BC.

## 1. Introduction

The use of neoadjuvant chemotherapy (NAC) in patients with breast cancer (BC) aims to decrease tumor size to promote breast conservation and convert unresectable tumors to resectable lesions [1,2]. In patients with pathologic complete response (pCR) after NAC, the absence of invasive cancer is associated with lower recurrence rates [3]. pCR is also associated with a favorable prognosis and increased rates of disease-free survival and overall survival (OS) [4]. In contrast, residual disease (RD) after NAC is associated with a poor prognosis. pCR is a surrogate for survival in BC, particularly in triple-negative and HER2-positive BC [5]. However, only 30–40% of patients with BC show pCR after NAC, and the predictive value of pCR varies depending on molecular tumor subtypes [6].

Exosomes are small extracellular vesicles (EVs) that participate in cell-to-cell communication in cancer and non-malignant cells [7,8]. Exosomes regulate various cancer-related signaling pathways and components of the tumor microenvironment [7,8]. Recent data suggest that exosomes also regulate drug resistance and tumor cell proliferation, invasion, and metastasis. As exosomes may carry ATP-binding cassette transporters, they can contribute to the efflux of drugs and/or their metabolites from tumor cells [8]. Exosomal cargo comprises proteins, mRNAs, microRNAs (miRNAs), lipids, and metabolites. Accumulating evidence indicates that exosomes can exacerbate tumor progression by transferring metabolites into the recipient cell, thereby preventing drug-mediated genotoxicity and promoting drug resistance [9,10,11,12]. However, exosomal metabolic signatures associated with differential NAC response in patients with BC remain unknown.

In this study, we identified metabolic pathways and metabolites that were differentially loaded in the plasma exosomes of patients with RD compared with those with pCR. Identifying exosomal metabolic signatures associated with RD or pCR may provide insight into the metabolic pathways that regulate NAC response in patients with BC and could help improve disease outcomes in non-responders.

## 2. Results

### 2.1. Isolation and Characterization of Exosomes from BC Patients with pCR or RD after NAC

**Figure 1:**
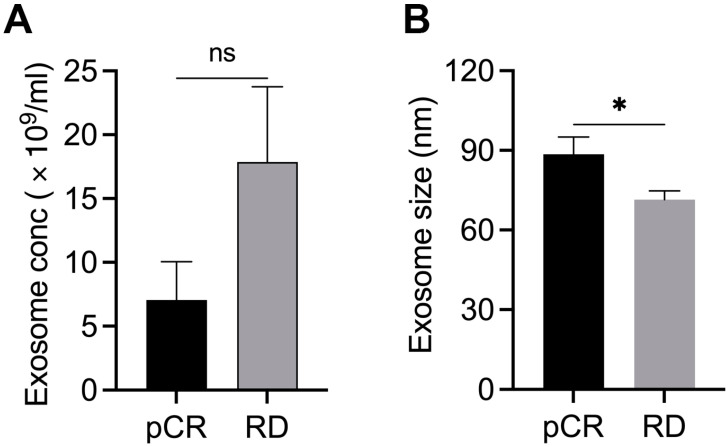
Characterization of exosomes in plasma samples from patients with BC showing pCR (*n* = 8) or RD (*n* = 8) after NAC. Bar graphs showing the concentration (**A**) and mean size (**B**) of exosomes in patients with pCR or RD after NAC. Bars indicate mean ± SEM. Unpaired two-tailed Student’s *t*-test with Welch’s correction was used to determine statistical significance (ns, non-significant; * *p* < 0.05).

The structural and functional heterogeneity in exosomal cargo may reflect the differential response to NAC in BC [7,13]. To understand any association between exosome concentration and metabolite cargo with NAC response, we isolated and characterized exosomes from the plasma of BC patients who showed pCR (*n* = 8) or RD (*n* = 8) after NAC. The concentration (number/mL) and size of purified exosomes were assessed using NTA. Interestingly, the concentration of exosomes was 2.52 fold higher, and their average size was 24% smaller (*p* < 0.05) in patients who exhibited RD than in those with pCR (Figure 1A,B). These data suggest that exosomal characteristics (size and concentration) might correlate with differential NAC outcomes in patients with BC.

### 2.2. Differentially Regulated Metabolic Pathways and Metabolites in Plasma Exosomes of BC Patients with RD and those with pCR after NAC

The exosomal cargo is rich in proteins, non-coding RNAs, and metabolites. Exosomes regulate intercellular communication by releasing their cargo into recipient cells [12,14]. Various studies have suggested that exosomal protein and miRNA signatures are associated with treatment outcomes in BC [7,15,16]. However, there is limited data on the relationship between metabolic signatures in exosomes and NAC response in BC. Thus, we performed a metabolomic analysis of exosomes from patients with pCR or RD after NAC to identify metabolic signatures associated with NAC outcomes (Figure S1, the significantly altered metabolites in pCR are shown in Supplementary Figure S2).

**Figure 2:**
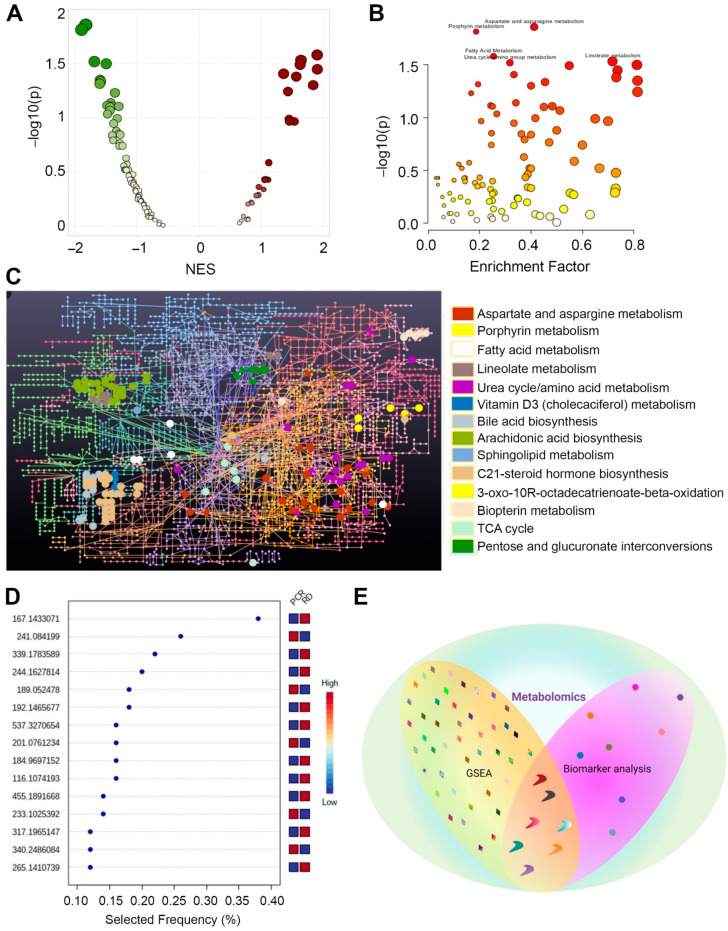
Differentially regulated metabolic pathways and metabolites in plasma exosomes of patients with pCR or RD after NAC. (**A**) Scatter plot showing metabolic pathways upregulated (maroon plots with positive NES) and downregulated (green plots with negative NES) in RD vs. pCR (analyzed using GSEA). The color intensity indicates the *p*-value, and the dot size indicates NES. (**B**) Scatter plot summarizing the pathways differentially enriched in patients with RD vs. patients with pCR after NAC; circles indicate matched pathways from user-uploaded data. The color intensity indicates the *p*-value, and the dot size indicates the enrichment score. (**C**) Network analysis showing metabolites in differentially regulated pathways in RD and pCR samples. Colors indicate different metabolic pathways, and circles indicate metabolites associated with a specific pathway. (**D**) Scatter plot showing the most important metabolites of a selected model ranked from most to least important (analyzed using biomarker analysis). (**E**) Venn diagram showing shared and differentially regulated metabolites identified by GSEA and biomarker analysis. Different shapes were used to differentiate the results of the two analyses. Colors are specific to metabolites that were differentially regulated in the two groups. NES, normalized enrichment score. This schematic was created using BioRender (Toronto, ON, Canada).

The GSEA of differentially regulated metabolic pathways and metabolites showed that various metabolic pathways were differentially regulated in plasma exosomes between patients with RD and those with pCR after NAC. Aspartate and asparagine metabolism, porphyrin metabolism, urea cycle, vitamin D3 metabolism, 3-oxo-10R octadecatrienoate beta-oxidation, biopterin metabolism, and TCA cycle were significantly downregulated in plasma exosomes of patients with RD compared to those with pCR after NAC (*p* ≤ 0.05; Figure 2A,B; Supplementary Table S2). In contrast, linoleate metabolism, bile acid biosynthesis, arachidonic acid metabolism, sphingolipid metabolism, C-21-steroid hormone biosynthesis, metabolism, and pentose glucuronate metabolism pathways were significantly upregulated in plasma exosomes of patients with RD (*p* ≤ 0.05). Network analysis of the enriched metabolic pathways revealed differentially regulated metabolites associated with these pathways (Figure 2C and Figure S2; Supplementary Tables S3 and S4).

Biomarker analysis was performed to identify commonalities and differences in the metabolites identified by GSEA. A ROC curve was plotted for the biomarker model (Figure S3). A comparison of the results from the GSEA and biomarker analysis demonstrated that thymidine, porphobilinogen, 1-methylhistidine, formiminoglutamic acid, carglumic acid, and azelaic acid were significantly downregulated in plasma exosomes of patients with RD compared to those with pCR after NAC (*p* ≤ 0.05; Figure 2D and Figure S2, Supplementary Table S5). CE5869 (its common name cannot be determined based on KEGG ID) was significantly upregulated in plasma exosomes of patients who showed RD after NAC (*p* ≤ 0.05; Figure 2E; Supplementary Table S5). An in-depth analysis of these common metabolites obtained using GSEA and biomarker analysis is warranted to understand the differential treatment outcomes after NAC in patients with BC. These data suggest that the metabolic signatures of plasma exosomes differ significantly between patients who exhibit pCR and those with RD after NAC.

### 2.3. Succinic Acid and L-Lactic Acid Are Significantly Upregulated in Plasma Exosomes of Patients with RD after NAC

**Figure 3:**
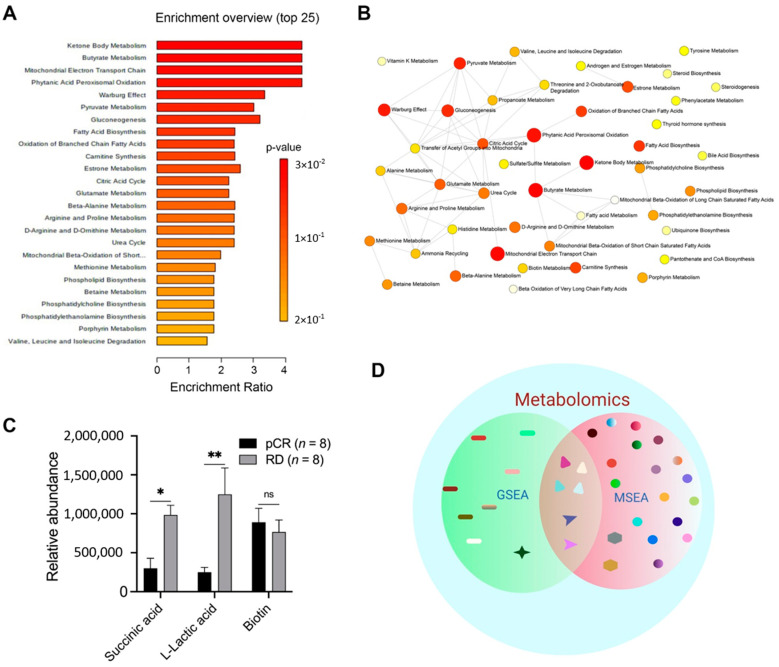
Succinic acid and L-lactic acid are significantly upregulated in plasma exosomes of patients with RD after NAC. Differentially regulated metabolic pathways and metabolites were identified using MSEA. (**A**) Network showing metabolic pathways differentially regulated in patients with RD or pCR after NAC. (**B**) Summary plot showing the top 25 differentially enriched metabolic pathways. Each node represents a metabolite set with its color based on its *p*-value and its size based on number hits to submitted query. Two metabolite sets are connected by an edge if the number of their shared metabolites is over 25% of the total number of their combined metabolite sets. (**C**) Bar graphs showing the levels of various metabolites in patients with RD and those with pCR. (**D**) Venn diagram showing common metabolites identified using GSEA and MSEA. Bars indicate mean ± SEM. Two-way ANOVA with Sidak’s multiple comparison test was used to determine statistical significance. ns, non-significant; * *p* < 0.05; ** *p* < 0.005; The scheme in (**D**) was created using Bi-oRender, Toronto, ON, Canada.

To further evaluate the metabolic pathways that were differentially regulated in the plasma exosomes of patients with RD and those with pCR, we performed MSEA, a complementary, direct, and more sensitive approach to GSEA. We identified various differentially regulated metabolic pathways (Figure 3A; Supplemetary Table S6). Among these, ketone body metabolism, butyrate metabolism, mitochondrial electron transport chain, phytanic acid peroxisomal oxidation, Warburg effect metabolism, pyruvate metabolism, gluconeogenesis, and fatty acid biosynthesis were significantly enriched in metabolites loaded in plasma exosomes of patients who exhibited RD compared to those who showed pCR after NAC (*p* ≤ 0.05; Figure 3B). Next, we extracted a list of metabolites associated with each of the differentially regulated pathways. Interestingly, in five out of seven differentially regulated metabolic pathways (ketone body metabolism, butyrate metabolism, mitochondrial electron transport chain, phytanic acid peroxisomal oxidation, and Warburg effect pathway), succinic acid was a common metabolite that was significantly upregulated in patients with RD compared with those with pCR (*p* ≤ 0.05; Figure 3C).

Additionally, in various enriched metabolic pathways, including Warburg effect metabolism, pyruvate metabolism, and gluconeogenesis, L-lactic acid was significantly (*p* ≤ 0.05) upregulated in plasma exosomes of patients with RD compared with those with pCR (Figure 3C). Biotin levels did not differ significantly between the two groups (Figure 3C), despite the involvement of these metabolites in various metabolic pathways. The analysis of the common metabolites identified through GSEA and MSEA (Figure 3D; Supplementary Tables S2 and S6) further supported the key importance of targeting succinic acid and L-lactic acid in patients with RD after NAC to improve their disease course.

## 3. Discussion

BC is a highly heterogeneous and complex disease, and patients with the same molecular subtype of BC often show differential treatment responses [15,16]. Drug resistance remains a key challenge in the treatment of BC [17,18]. Exosomes play a crucial role in BC development and progression, and their role in drug resistance is becoming increasingly evident [19,20]. Exosomes regulate intercellular communication and modulate the tumor microenvironment [7,21]. Tumor-derived exosomes can also promote chemoresistance by decreasing the availability of chemotherapeutic drugs and delivering functional cargo that triggers cancer cell survival [13,22]. Data by Lv M.M. et al., (2014) [13] have suggested that exosomes released by drug-resistant cells had elevated levels of P-glycoprotein (P-gp), (an important transmembrane protein involved in drug efflux mechanisms). In contrast, exosomes from sensitive cells expressed low levels of P-gp [13]. Thus, exosomes may help predict and monitor treatment response in patients with BC. Particularly in patients receiving NAC, early detection of RD is crucial for optimizing treatment strategies to promote the development of pCR. Studying the role of exosomal concentration and cargo in chemoresistance could provide new diagnostic and prognostic biomarkers and therapeutic targets for BC.

Many studies have investigated the role of exosomal proteins and miRNAs in drug resistance in BC [7,15,16]. Exosomal cargo is complex and contains metabolites [23,24] that may modulate drug response in cancer [25]. Exosomes also act as a source of metabolite cargo, enabling metabolic reprogramming in cancer. The crosstalk between metabolic circuits and signaling pathways plays a central role in cancer progression [26,27,28]. Metabolic rewiring has emerged as a key mechanism for acquired drug resistance in BC [29,30]. Oncometabolites in exosomal cargo are transferred to recipient cancer cells and influence treatment response by modulating cell metabolism. Although a thorough understanding of the exosomal metabolites may hold potential diagnostic value in cancer, the metabolome of exosomes has not been widely studied. In this study, we performed an in-depth metabolomics analysis of exosomes isolated from the plasma of BC patients with pCR or RD after NAC. Our metabolomics data suggest that metabolic signatures in plasma-derived exosomes could discriminate NAC responders (i.e., patients with pCR) from non-responders (i.e., patients with RD), supporting the strong link between exosomes mediated metabolic rewiring and could be extrapolated to drug resistance in BC.

Unsaturated fatty acids distort the hydrophobic chain of lipid membranes, leading to lose membrane packaging and increased membrane fluidity. In contrast, saturated fatty acids make the membrane more rigid and decrease membrane fluidity [31], affecting drug uptake [32]. A comparison of plasma exosomal metabolites between patients with pCR and those with RD after NAC revealed a significant enrichment of fatty acid biosynthesis in patients with RD. Increased levels of fatty acids in exosomes from patients with RD may contribute to high levels of lipid saturation and low membrane fluidity, leading to drug resistance due to reduced drug uptake. Thus, targeting fatty acid biosynthesis pathway may improve disease outcomes in patients with RD after NAC.

The citrate acid cycle pathway was also enriched in plasma exosomes of the patients with RD. Succinate, a central metabolite in the citrate acid cycle, has been implicated in drug resistance in cancer. Increased levels of succinate in tumors cause pseudohypoxia, which stabilizes hypoxia inducible factor-1 (HIF-1) in normoxic conditions. Pseudohypoxia promotes cell survival, proliferation, and angiogenesis. By stabilizing HIF-1, succinate promotes the upregulation of several drug efflux transporters, leading to multidrug resistance [30,32,33]. Succinic acid was a common metabolite among various metabolic pathways that were upregulated in plasma exosomes from patients with RD after NAC, suggesting that targeting succinic acid using commercially available pharmacological inhibitors (e.g., compound 968 and CB-839) could improve disease course in patients with RD.

BC cells often experience anaerobic conditions, and glucose is catabolized anaerobically via glycolysis to generate lactate rather than being converted into carbon dioxide via the citrate acid cycle [33]. Lactate has emerged as a critical player in BC carcinogenesis, and elevated lactate levels are associated with poor prognosis. Dong et al. [34] showed that lactic acid-induced MRP1 expression hindered the genotoxic and apoptotic effects of chemotherapeutic drugs by enhancing drug efflux. In this study, we found that lactic acid was a common metabolite among various metabolic pathways that were enriched in exosmes of patients with RD.

The metabolic pathways identified in this study have been previously implicated in drug resistance in cancer [28,30,31]. However, to the best of our knowledge, this is the first study to evaluate the relationship between exosomal metabolites and NAC response in patients with BC. The metabolic signatures identified in this study could be used to stratify patients with BC before the administration of NAC to predict treatment response and improve disease outcomes. Although these data provide a foundation for identifying metabolic signatures in patients with RD and pCR after NAC, the main caveat of the present study is the small cohort size and lack of in vitro validation data. Further validation of these findings in large cohorts of patients treated with NAC is warranted. Moreover, an evaluation of the role of the BC subtype and type of chemotherapy could provide valuable information regarding the differential metabolomic signatures associated with response to NAC in patients with BC. Lastly, one of the major limitations of the present study is that we analyzed total exosomes in plasma with vesicles contributed by almost all cell types, including BC cells. Therefore, the exosome characterization and cargo analyses presented here are not specific to BC. Any metabolic analyses in plasma exosomes could only serve as a biomarker of association with the treatment outcome pCR or RD; and very remotely an indirect measure of BC tumor or its microenvironment components. In future studies, there is a need to first enrich BC cells-derived exosomes from total plasma exosomes towards the development of more specific biomarkers and the identification of potential therapeutic targets.

In conclusion, our data strongly suggest that metabolic rewiring is evident in patients with RD after NAC. We identified exosomal metabolic signatures associated with RD or pCR after NAC in patients with BC. These exosomal measures could serve as a potentially useful biomarker in the clinic to predict BC response to NAC.

## 4. Materials and Methods

### 4.1. Patients

In this study, we included adult (≥18 years old) female patients with histologically confirmed stage I–III BC diagnosed at Emory University Hospital, and for whom NAC was recommended (Table S1). Pre-NAC core biopsy and plasma specimens were available for all the patients enrolled in the study. Patients who received ≥ 4 doses/week of non-aspirin nonsteroidal anti-inflammatory drugs (NSAIDs), aspirin (>81 mg/day), or systemic corticosteroids 14 days prior to enrollment were excluded from the study because these medications could affect plasma cytokines. Pathologic response to NAC was assessed using the MD Anderson Residual Cancer Burden (RCB) index [35]. pCR was defined as RCB = 0. RD was classified as RCB-I (RCB < 1.36), RCB-II (1.36 < RCB < 3.28), and RCB-III (RCB > 3.28). Blood samples (pre-NAC) were collected from patients who showed pCR and RD (*n* = 8 each), and exosomes were isolated for further analysis. All patients provided written informed consent. This study was approved by the Institutional Review Board (IRB) of Emory University. All procedures were conducted in accordance with the guidelines of the Emory IRB and Winship Clinical and Translational Review Committee (CRTC).

### 4.2. Exosome Isolation

Blood samples were collected in EDTA-containing vacutainer tubes using standard sterile venipuncture techniques. Plasma was recovered by centrifugation of whole blood at 1000× *g* for 10 min at 4 °C. Plasma was removed, aliquoted into siliconized polypropylene tubes, and stored at −80 °C until batch assay. Total exosomes were isolated from plasma by ultracentrifugation and used for nanoparticle tracking analysis (NTA) to characterize their concentration (number/mL), size distribution, and exosomal content. Plasma samples were diluted (1:1) with PBS and sequentially centrifuged at 500× *g*, 2000× *g* and 10,000× *g* to remove dead cells, debris, and larger-sized vesicles. The supernatant was passed through a 0.22 μm filter and ultracentrifuged at 100,000 × *g* for 120 min. The resulting pellets containing exosomes (small EVs) were collected. Exosome concentration (number/mL) and size distribution were analyzed using a NanoSight NS300 system (Malvern Instruments Ltd., Worcestershire, UK).

### 4.3. Isolation of Metabolites from Exosomes

Metabolite analysis in plasma exosomes was performed at the Translational Science Lab at the College of Medicine, Florida State University, Tallahassee, Florida, United States. A high-efficiency liquid–liquid extraction method was employed to extract metabolites from exosomes. Before metabolite extraction, three freeze-thaw cycles (snap freezing in liquid nitrogen followed by thawing on ice for 5 min) were performed to accelerate the release of metabolites from exosomes. Freeze-thaw cycles were followed by the addition of 300 µL methanol to 100 µL of exosomes for metabolite extraction. Samples were incubated with shaking for 15 min at room temperature to improve metabolite extraction. After incubation, samples were centrifuged at 21,000× *g* (4 °C) for 10 min, and the supernatants were transferred into a fresh tube. The pellets were resuspended in 300 µL of water and incubated with shaking for 15 min at room temperature. Samples were centrifuged at 21,000× g (4 °C) for 10 min, and the supernatants were collected. Extracted metabolites were dried in a SpeedVac, resuspended in 20 µL sample loading buffer (0.1% formic acid), and centrifuged at 21,000× g (4 °C) for 10 min. Supernatants were collected in glass vials.

### 4.4. Liquid Chromatography-Mass Spectrometry(LC-MS)

LC-MS was performed using an externally calibrated high-resolution electrospray tandem mass spectrometer (Thermo Q Exactive HF, Thermo Scientific, Waltham, MA, USA) in conjunction with Dionex UltiMate 3000 RSLCnano System (Thermo Scietific, Waltham, MA, USA). Samples (2 μL) were aspirated into 50 μL loops and loaded onto trap columns (Thermo µ-Precolumn 5 mm, with nanoViper tubing 30 µm internal diameter × 10 cm, Waltham, MA, USA). The flow rate was set to 300 nL/min for separation on the analytical column (Acclaim PepMap RSLC 75 μM × 15 cm nanoViper). The mobile phase A was composed of 99.9% H_2_O (EMD Omni Solvent) and 0.1% formic acid, and the mobile phase B was composed of 99.9% acetonitrile and 0.1% formic acid. A 30-min linear-gradient (from 3% to 60%) was performed. The eluent was directly nanosprayed onto a mass spectrometer. During chromatographic separation, the mass spectrometer was operated in a data-dependent mode using a Thermo Excalibur 3.1.66 (Thermo Scientific, Waltham, MA, USA). Survey scan was acquired in profile mode at 120,000 resolution (75–1000 *m*/*z*). In total, 20 data-dependent MS/MS scans per full scan were acquired in centroid mode at 15,000 resolution. Ions with charge >5 or unassigned charges were excluded. A 15-s dynamic exclusion window was used. All measurements were performed at room temperature. Raw files were analyzed using Compound Discoverer 3.0 and Untargeted Metabolomics workflow to identify differences between samples, as well as group compounds, and predict elemental compositions for all compounds. Compounds were identified using mzCloud (ddMS2) and ChemSpider (formula or exact mass) databases. The mzLogic algorithm was employed to rank ChemSpider results and map compounds to biological pathways using Metabolika. All statistical analyses, including differential analysis (*t*-test or ANOVA) and determination of *p*-values, adjusted *p*-values, ratios, and fold changes, were performed as part of this workflow.

### 4.5. Metabolomics Data Analysis

Metabolomic data (peak intensities and concentrations) were uploaded to the online portal MetaboAnalyst for analysis. Gene set enrichment analysis (GSEA), biomarker analysis, and metabolite set enrichment analysis (MSEA) were performed to determine differential metabolic signatures associated with response to NAC in patients with BC.

### 4.6. GSEA

The knowledge base for GSEA consists of five genome-scale metabolic models obtained from the original Python implementation which have either been manually curated or downloaded from BioCyc, an expanded library of 21 organisms derived from KEGG metabolic pathways, and 10 distinct metabolite set libraries. The selected library was hsa_m_fn. A total of 13,366 *m*/*z* features were found in the uploaded data. The instrument’s mass accuracy was 5 ppm, with negative ion mode. The range of *m*/*z* peaks was trimmed to 50–2000; 0 features were trimmed. A total of 13,366 input *m*/*z* features were retained for further analysis. The *p*-value cut-off was 0.25%. Default top 10% peaks were selected. The output of the GSEA consisted of a table of results containing ranked pathways that were enriched in the user-uploaded data. The table included the total number of hits (all and expected), raw *p*-values, and adjusted *p*-values.

### 4.7. Biomarker Analysis

Biomarker analysis involved data processing, biomarker selection, performance evaluation, and model creation. For this analysis, a peak intensity table was uploaded. Data were not filtered. Zero or missing values were replaced by 1/5 of the minimum positive value for each variable. During normalization, ratios between metabolite concentrations were calculated. Ratios between two metabolite concentrations may provide more information than the two metabolite concentrations separately. Ratios between all possible metabolite pairs were computed, and the top-ranked ratios (based on *p*-values) were selected for further analysis. Multivariate exploratory receiver operating characteristic curve analysis was performed to evaluate the performance of biomarker models created through automated important feature identification.

### 4.8. MSEA

For this analysis, a metabolite concentration table was uploaded. Human metabolome database (HMDB) identifiers were used for the analysis. Metabolites with missing or negative values were excluded from the analysis. Quantitative enrichment analysis (QEA) was performed using the package globaltest [36]. A generalized linear model was used to estimate the Q-statistic for each metabolite set as the average of the Q-statistic for each of the metabolites in the set.

### 4.9. Statistical Analysis

Statistical significance was determined using a two-tailed Student’s *t*-test with Welch’s correction or two-way analysis of variance (ANOVA) with Sidak’s test for multiple comparisons. Data were expressed as mean ± standard error mean (SEM). Statistical analyses were performed using GraphPad Prism 9 (San Diego, CA, USA)

## Data Availability

Not applicable.

## Supplementary Materials

The Supplementary Materials are available online at https://www.mdpi.com/article/10.3390/ijms23105324/s1.
