# Supplementary material for: Exosomal Metabolic Signatures Are Associated with Differential Response to Neoadjuvant Chemotherapy in Patients with Breast Cancer
Source: Int J Mol Sci. 2022 May 10;23(10):5324. doi: 10.3390/ijms23105324 (PMC9141543; doi:10.3390/ijms23105324)
Supplement: Supplementary file 1 [file ijms-23-05324-s001.zip › ijms-1659214-supplementary.pdf]

Sr. No.	pCR or RD	cT	cN	Clinical Stage	Nottingham Histologic Grade	ER	PR	HER2	Chemotherapy Type	Chemo Cycles	Chemo Start Date	Chemo Finish Date
**1**	pCR	cT2	cN1	IIB	G2	+	-	+	AC+Taxol, HER	12	12/19/2014	5/15/2015
**2**	pCR	cT3	cN1	IIIA	high grade	-	-	-	AC+Taxol	12	8/22/2017	12/27/2017
**3**	pCR	cT4d	cN3c	IIIC	G3	+	-	Equivocal	THP x 6, Taxol x 12	18	7/28/2017	10/20/2017
**4**	pCR	cT2	cN1	IIIB	G3	-	-	-	ddACx3 (discontinued due to side effects), Taxol x 12	12	9/12/2017	1/23/2018
**5**	pCR	cT2	cN2	IIIA	G2	+	+	-	AC+Taxol	8	4/11/2014	7/18/2014
**6**	pCR	cT2	cN1	IIB	G3	+	-	+	TCHP, HER	6	4/25/2017	8/15/2017
**7**	pCR	cT2	cN0	IIA	G2-G3	-	-	-	AC+Taxol	4	4/21/2017	6/2/2017
**8**	pCR	cT3	cN0	IB	G3	+	+	+	TCHP (4th cycle w/out taxotere), HER	4	8/28/2017	11/13/2017
**9**	RD	cT2	cN1	IIB	G2	+	+	+	TCHP	6	10/31/2014	2/27/2015
**10**	RD	cT2	cN0	IIA	G3	-	-	-	AC+Taxol	4	9/15/2017	12/18/2017
**11**	RD	cT4b	cN2	IIIB	G3	-	-	+	TPHx4 and FECx3	7	1/16/2014	5/22/2014
**12**	RD	cT4b	cN3c	IIIC	unknown	-	-	-	TC, ddAC	4	8/4/2015	12/28/2015
**13**	RD	cT2	cN0	IIA	G3	+	-	+	TCPH, (HER&Perjeta only 2 cycles due to neuropathy)	6	12/31/2015	4/14/2016
**14**	RD	cT1c	cN0	IA	G2	-	-	Equivocal	AC+Taxol	8	12/14/2017	3/29/2018
**15**	RD	cT2	cN0	IIB	G3	-	-	-	AC+Taxol	8	2/2/2018	6/19/2018
**16**	RD	cT2	cN1	IIIB	G3	-	-	-	AC+Taxol	4	5/18/2018	8/10/2018

Table S1: Details of the cohort

Metabolic pathways	Pathway Total	Hits	P-value	NES
Aspartate and asparagine metabolism	114	47	0.014	-1.815
Porphyrin metabolism	43	8	0.015	-1.883
Fatty acid metabolism	63	16	0.026	1.903
Linoleate metabolism	46	33	0.029	1.643
Urea cycle/amino group metabolism	85	27	0.03	-1.677
Vitamin D3 (cholecalciferol) metabolism	16	13	0.032	-1.486
Bile acid biosynthesis	82	45	0.032	1.629
Arachidonic acid metabolism	95	70	0.036	1.906
Sphingolipid metabolism	3	1	0.039	1.364
C21-steroid hormone biosynthesis and metabolism	112	82	0.042	1.567
3-oxo-10R-octadecatrienoate beta-oxidation	27	22	0.045	-1.586
Biopterin metabolism	22	10	0.046	-1.574
TCA cycle	31	6	0.048	-1.59
Pentose and glucuronate interconversions	15	6	0.05	1.839
Putative anti-Inflammatory metabolites formation from EPA	27	22	0.057	1.434
3-Chloroacrylic acid degradation	6	1	0.059	-1.346
Glycine, serine, alanine and threonine metabolism	88	30	0.072	-1.414
Starch and sucrose metabolism	33	8	0.077	-1.474
Ascorbate (Vitamin C) and aldarate metabolism	29	14	0.078	-1.39
Benzoate degradation via CoA ligation	4	1	0.078	-1.284
Hexose phosphorylation	20	9	0.079	-1.426
Galactose metabolism	41	21	0.086	-1.427
Alanine and aspartate metabolism	30	8	0.092	-1.466
Carnitine shuttle	72	30	0.101	-1.309
Pyruvate metabolism	20	13	0.103	1.452
Limonene and pinene degradation	10	7	0.108	1.537
Phytanic acid peroxisomal oxidation	34	7	0.108	1.456
Arginine and proline metabolism	45	15	0.113	-1.366
1- and 2-methylnaphthalene degradation	4	2	0.132	-1.325
Valine, leucine and isoleucine degradation	65	16	0.141	-1.287
Tyrosine metabolism	160	58	0.143	-1.261
Glutamate metabolism	15	6	0.145	-1.39
N-glycan degradation	16	6	0.161	-1.375
Butanoate metabolism	34	16	0.172	-1.269
Vitamin H (biotin) metabolism	5	3	0.182	-1.276
Xenobiotics metabolism	110	27	0.182	-1.225
Tryptophan metabolism	94	35	0.236	-1.168

Androgen and estrogen biosynthesis and metabolism	95	54	0.259	1.125
Sialic acid metabolism	107	17	0.268	-1.15
Phosphatidylinositol phosphate metabolism	59	7	0.277	-1.205
Fructose and mannose metabolism	33	13	0.302	-1.124
Aminosugars metabolism	69	9	0.302	-1.191
Vitamin K metabolism	3	2	0.302	-1.178
Carbon fixation	10	4	0.304	-1.161
Prostaglandin formation from arachidonate	78	57	0.333	-1.059
Propanoate metabolism	31	12	0.343	-1.123
Pentose phosphate pathway	37	14	0.368	1.077
Vitamin B1 (thiamin) metabolism	20	4	0.37	1.055
Di-unsaturated fatty acid beta-oxidation	26	1	0.373	1.115
Fatty acid oxidation	35	1	0.373	-1.079
Fatty acid oxidation, peroxisome	28	1	0.373	-1.097
Nucleotide sugar metabolism	7	1	0.373	-1.101
Saturated fatty acids beta-oxidation	36	5	0.375	1.058
C5-branched dibasic acid metabolism	10	1	0.392	-1.048
Vitamin B3 (nicotinate and nicotinamide) metabolism	28	7	0.4	-1.08
Selenoamino acid metabolism	35	5	0.403	-1.05
Vitamin B6 (pyridoxine) metabolism	11	2	0.415	-1.043
Proteoglycan biosynthesis	27	1	0.431	-1.008
Glutathione metabolism	19	4	0.435	0.965
Methionine and cysteine metabolism	94	18	0.447	-0.993
Drug metabolism - other enzymes	31	12	0.457	1.042
Alkaloid biosynthesis II	10	4	0.464	-1.018
D4&E4-neuroprostanes formation	37	27	0.47	-0.956
Histidine metabolism	33	8	0.477	-1.012
Pyrimidine metabolism	70	18	0.513	-0.952
Prostaglandin formation from dihomo gamma-linoleic acid	11	8	0.514	0.96
Vitamin A (retinol) metabolism	67	37	0.517	0.9614
N-Glycan biosynthesis	48	4	0.522	0.9252
Dynorphin metabolism	8	2	0.528	-0.972
Drug metabolism - cytochrome P450	53	30	0.536	-0.97
Omega-6 fatty acid metabolism	55	3	0.564	-0.982
Glycosphingolipid biosynthesis - globoseries	16	2	0.566	-0.945
Glycolysis and gluconeogenesis	49	17	0.578	-0.921
Beta-Alanine metabolism	20	7	0.585	-0.93
Glycosphingolipid biosynthesis - ganglioseries	62	6	0.6	0.8434
Hyaluronan metabolism	8	2	0.612	0.8567
Purine metabolism	80	19	0.622	-0.88
Omega-3 fatty acid metabolism	39	6	0.625	0.7906

Lysine metabolism	52	20	0.634	-0.884
Parathion degradation	6	2	0.679	-0.828
Vitamin B9 (folate) metabolism	33	2	0.679	-0.868
Glycosphingolipid metabolism	67	17	0.732	-0.794
Squalene and cholesterol biosynthesis	55	29	0.735	-0.836
CoA catabolism	7	2	0.774	-0.749
Nitrogen metabolism	6	1	0.804	-0.828
Chondroitin sulfate degradation	37	3	0.809	0.7184
Heparan sulfate degradation	34	3	0.809	0.7184
Glyoxylate and dicarboxylate metabolism	12	5	0.823	-0.754
Vitamin B12 (cyanocobalamin) metabolism	9	1	0.824	-0.814
Vitamin E metabolism	54	34	0.829	-0.775
Leukotriene metabolism	92	40	0.852	0.7942
Glycerophospholipid metabolism	156	38	0.867	0.7724
Fatty acid activation	74	35	0.867	0.7961
Keratan sulfate degradation	68	4	0.875	-0.705
Ubiquinone biosynthesis	10	3	0.894	0.6801
Vitamin B5 - CoA biosynthesis from pantothenate	12	3	0.909	-0.631
De novo fatty acid biosynthesis	106	20	0.936	0.6545
Polyunsaturated fatty acid biosynthesis	21	2	0.962	-0.64
Lipoate metabolism	8	4	0.982	-0.574

Table S2. Metabolic pathways differentially regulated in BC patients with RD or pCR after NAC. Pathways were identified using GSEA (corresponding to **Figure 2 A-C**). Pathways in red are significantly enriched in patients with RD compared to those with pCR ($p \leq 0.05$). Pathways in green are common hits from GSEA and MSEA. Pathways with positive NES were enriched in the RD group, while pathways with negative NES were enriched in the pCR group. NES, normalized enrichment score.

**Pathway name**	**KEGG ID of metabolites**	**Common name metabolite**
Aspartate and asparagine metabolism	C00148	L-proline
	C00437	N-acetylornithine
	C01029	N8-acetylspermidine
	CE1661	NA
	C03440	cis-4-hydroxy-D-proline
	C00232	Succinic acid semialdehyde
	C05829	Carglumic acid
	C01035	4-guanidinobutanoic acid
	C01239	N-acetyl-b-glucosaminylamine
	CE1556	NA
	CE1935	NA

	CE1936	NA
	CE1939	NA
	CE1938	NA
	C03740	Gamma-glutamylalanine
	C00555	NA
	C00077	Ornithine
	C02567	N1-acetylspermine
	C05572	4-oxoglutaramate
	C00086	Urea
	C02356	L-alpha-aminobutyric acid
	C00487	Malonyl-carnitine
	C05931	N-succinyl-L-glutamate
	C05932	N_2-succinyl-L-glutamic acid 5-semialdehyde
	C00064	L-glutamine
	C00109	3-methyl pyruvic acid
	C02571	L-acetylcarnitine
	C03078	4-guanidinobutanamide
	C00986	1,3-diaminopropane
	CE1059	NA
	C00989	Gamma hydroxybutyric acid
	C02630	2-hydroxyglutaric acid
	C00750	Spermine
	C01157	4-hydroxyproline
	C00612	N1-acetylspermidine
	C03413	N1,N12-Diacetylspermine
	C03415	N_2-succinyl-L-ornithine
	C00327	Citrulline
	C01165	L-glutamic gamma-semialdehyde
	CE5588	NA
	C02946	4-acetamidobutanoic acid
	C02714	N-acetylputrescine
	CE1940	NA
	C00033	Acetic acid
	CE1943	NA
	C00334	Gamma-aminobutyric acid
	C00047	L-lysine
Porphyrin metabolism	C15670	Chenodeoxyglycocholoyl-CoA
	C00430	5-aminolevulinic acid
	C00072	Ascorbate
	C00931	Porphobilinogen
	C01079	Protoporphyrinogen IX

	C00500	Biliverdin
	C00425	NA
	C00486	Bilirubin
Fatty Acid Metabolism	C02571	L-acetylcarnitine
	hdcea	NA
	C01607	Phytanic acid
	C00318	L-carnitine
	C02679	Dodecanoic acid
	ocdcya	NA
	C05265	3-oxodecanoyl-CoA
	lneldc	NA
	C06424	Myristic acid
	ttdcea	NA
	C00249	Palmitic acid
	C01595	Linoleic acid
	ocdcea	NA
	C01530	Stearic acid
	C00187	Cholesterol
	dmnoncrn	NA
Linoleate metabolism	CE2061	NA
	C04230	LysoPC(18:1(9Z))
	CE6504	NA
	CE6415	NA
	CE2306	NA
	C01041	Ascorbic acid
	CE6506	NA
	CE2576	NA
	C04717	13-L-hydroperoxylinoleic acid
	C08261	Azelaic acid
	CE5922	NA
	CE5920	NA
	CE2006	NA
	CE2577	NA
	C00072	Ascorbate
	C00157	PC(16:0/16:0)
	CE5526	NA
	C14825	9,10-epoxyoctadecenoic acid
	C14826	12,13-EpOME
	C14827	9(S)-HPODE
	CE2047	NA
	CE2049	NA
	CE2305	NA
	CE2304	NA

	CE2303	NA
	CE5528	NA
	CE6502	NA
	C06426	Gamma-linolenic acid
	CE5527	NA
	C14762	13S-hydroxyoctadecadienoic acid
	C01595	Linoleic acid
	C14765	13-oxoODE
	C01601	Pelargonic acid
Urea cycle/amino group metabolism	C00148	L-Proline
	C00021	S-adenosylhomocysteine
	C00101	Tetrahydrofolic acid
	C00327	Citrulline
	C02946	4-acetamidobutanoic acid
	C00750	Spermine
	C04692	2-(3-carboxy-3-(methylammonio)propyl)-L-histidine
	C00437	N-acetylornithine
	C02735	2-hydroxyphenethylamine
	C05313	3-hexaprenyl-4-hydroxy-5-methoxybenzoic acid
	C00555	NA
	C00300	Creatine
	C00179	Agmatine
	C00213	Sarcosine
	C00033	Acetic acid
	C00334	Gamma-aminobutyric acid
	C03711	N-methylphenylethanolamine
	C05198	5'-deoxyadenosine
	C01137	S-adenosylmethioninamine
	C00086	Urea
	C00242	Guanine
	C01165	L-glutamic gamma-semialdehyde
	C05200	3-hexaprenyl-4,5-dihydroxybenzoic acid
	C01449	Queuine
	C00788	Epinephrine
	C00547	Norepinephrine
	C01602	L-ornithine monochlorohydrate/ornithine

Vitamin D3 (cholecalciferol) metabolism	CE5079	NA
	CE6027	NA
	C01673	Calcitriol
	C01164	7-Dehydrocholesterol
	C05443	Vitamin D3
	CE2206	NA
	CE2207	NA
	CE2204	NA
	CE2205	NA
	CE2202	NA
	CE2203	NA
	CE2201	NA
	CE1337	NA
Bile acid biosynthesis	C15610	27-hydroxy-cholesterol
	C05463	Taurodeoxycholic acid
	CE1279	NA
	C05465	Tetrahydrocortisol
	C05444	3 alpha,7 alpha,26-trihydroxy-5beta-cholestane
	C05445	3a,7a-dihydroxy-5b-cholestan-26-al
	C05446	27-deoxy-5b-cyprinol
	C04722	3a,7a,12a-trihydroxy-5b-cholestanoic acid
	C03594	7a-hydroxycholesterol
	CE5530	NA
	CE5560	NA
	C01921	Glycocholic acid
	C05462	Chenodeoxyglycocholic acid
	C03990	NA
	C05451	7a-hydroxy-5b-cholestan-3-one
	CE4874	NA
	CE1277	NA
	C05458	7a,12a-dihydroxy-5a-cholestan-3-one
	CE1272	NA
	C05453	7a,12a-dihydroxy-5b-cholestan-3-one
	C05452	3a,7a-dihydroxy-5b-cholestane
	C15520	7alpha,25-dihydroxycholesterol
	C05457	7a,12a-dihydroxy-cholestene-3-one
	C05454	5-b-cholestane-3a,7a,12a-triol

	C05466	Chenodeoxycholic acid glycine conjugate
	C17339	4-cholesten-7alpha,12alpha-diol-3-one
	C00695	Cholic acid
	C17335	3beta,7alpha-dihydroxy-5-cholestenoate
	C17337	7alpha-hydroxy-3-oxo-4-cholestenoate
	C17336	7 alpha,26-Dihydroxy-4-cholesten-3-one
	C17331	7 alpha,24-Dihydroxy-4-cholesten-3-one
	C17333	3 beta-Hydroxy-5-cholestenoate
	C17332	7alpha,25-Dihydroxy-4-cholesten-3-one
	C15518	7alpha,24S-Dihydroxycholesterol
	CE1278	NA
	C02528	Chenodeoxycholic acid
	C04483	Deoxycholic acid
	C02592	Lithocholyltaurine
	C15517	Hyodeoxycholic acid
	C00163	Propionic acid
	C00187	Cholesterol
	C15519	25-Hydroxycholesterol
	C06341	7-a,27-Dihydroxycholesterol
	C13550	24-Hydroxycholesterol
	C06340	27-Hydroxycholesterol
Arachidonic acid metabolism	12harachd	NA
	CE7228	NA
	C14772	5,6-DHET
	C05356	5(S)-Hydroperoxyeicosatetraenoic acid
	C06315	Lipoxin B4
	C06314	Lipoxin A4
	C04741	Prostaglandin E1
	C04742	15(S)-HETE
	CE5705	NA
	C05966	15(S)-HPETE
	C05965	12(S)-HPETE
	C00157	PC(16:0/16:0)
	CE6248	NA
	CE6246	NA

	CE6247	NA
	C00427	Prostaglandin H_2
	C14749	19(S)-HETE
	C14748	20-hydroxyeicosatetraenoic acid
	CE7231	NA
	CE7243	NA
	CE7244	NA
	CE7096	NA
	CE7097	NA
	CE6251	NA
	CE6250	NA
	C14778	16(R)-HETE
	C14779	9S-HETE
	arachd	NA
	C14774	11,12-DiHETrE
	C14775	14,15-DiHETrE
	C14770	11,12-EpETrE
	C14771	14,15-Epoxy-5,8,11-eicosatrienoic acid
	C04654	(13E)-11a-hydroxy-9,15-dioxoprost-13-enoic acid
	C14773	8,9-DiHETrE
	CE5815	NA
	CE1450	NA
	C06427	Alpha-linolenic acid
	C14769	8,9-epoxyeicosatrienoic acid
	C14768	5,6-epoxy-8,11,14-eicosatrienoic acid
	C04843	NA
	C01312	Prostaglandin I2
	CE5178	NA
	C04849	NA
	C02198	Thromboxane A2
	C14813	11H-14,15-EETA
	C14781	15H-11,12-EETA
	C14782	11,12,15-THETA
	C00584	Prostaglandin E2
	C14814	11,14,15-THETA
	CE5661	NA
	CE5663	NA
	CE5662	NA
	leuktrC4	NA
	C00909	Leukotriene A4
	CE2568	NA

	CE2084	NA
	CE0347	NA
	C02165	Leukotriene B4
	C00696	Prostaglandin D2
	CE7172	NA
	CE2569	NA
	CE2567	NA
	CE2566	NA
	CE2565	NA
	leuktrE4	NA
	C00219	Arachidonic acid
	CE7234	NA
	C00639	Prostaglandin F2a
	C04805	5-HETE
	CE2449	NA
Sphingolipid metabolism	C00195	Ceramide
C21-steroid hormone biosynthesis and metabolism	C15610	27-hydroxy-cholesterol
	andrstndn	NA
	chsterols	NA
	C01227	Dehydroepiandrosterone
	C05485	21-hydroxypregnenolone
	C05487	17alpha,21-dihydroxypregnenolone
	C05488	Cortexolone
	C05489	11b,17a,21-trihydroxypreg-nenolone
	C00535	Testosterone
	C00951	Estradiol
	C04518	17-alpha,20-alpha-dihydroxypregn-4-en-3-one
	C05499	(20R)-17alpha,20-dihydroxycholesterol
	C05498	11b-hydroxyprogesterone
	C05497	21-deoxycortisol
	C03205	Deoxycorticosterone
	C05490	11-dehydrocorticosterone
	C15519	25-hydroxycholesterol
	CE1347	NA
	C05469	17a,21-dihydroxy-5b-pregnane-3,11,20-trione
	CE1349	NA
	C03917	Dihydrotestosterone
	C01124	18-hydroxycorticosterone

	C03748	16alpha-hydroxyprogesterone
	C05141	Estriol
	CE1352	NA
	C00410	Progesterone
	C00762	Cortisone
	C00468	Estrone
	C05471	Dihydrocortisol
	C05470	NA
	C05473	11b,21-dihydroxy-3,20-oxo-5b-pregnan-18-al
	C05472	NA
	C05475	11b,21-dihydroxy-5b-pregnane-3,20-dione
	CE1350	NA
	CE1353	NA
	C05476	Tetrahydrocorticosterone
	C05479	NA
	C05478	3a,21-dihydroxy-5b-pregnane-11,20-dione
	5adtststerones	NA
	C00084	Aldehyde
	tststerones	NA
	C03772	Etiocholanedione
	C00486	Bilirubin
	C06341	7-a,27-dihydroxycholesterol
	C00370	Sterol
	C05474	3a,11b,21-trihydroxy-20-oxo-5b-pregnan-18-al
	andrstrnglc	NA
	C05444	3 alpha,7 alpha,26-trihydroxy-5beta-cholestane
	C05446	27-deoxy-5b-cyprinol
	C01780	Aldosterone
	C05477	NA
	5adtststeroneglc	NA
	C05452	3a,7a-dihydroxy-5b-cholestane
	C15520	7alpha,25-dihydroxycholesterol
	C05454	5-b-cholestane-3a,7a,12a-triol
	C04555	Dehydroepiandrosterone sulfate
	dhea	NA
	prgnlones	NA
	C02373	3a,7b,12a-trihydroxy-5a-Cholanoic acid
	C01176	17-hydroxyprogesterone
	CE5888	NA

	C00187	Cholesterol
	C05501	20a,22b-dihydroxycholesterol
	C05500	20alpha-hydroxycholesterol
	C05502	22b-hydroxycholesterol
	C00280	Androstenedione
	estradiolglc	NA
	ahandrostanglc	NA
	hestratriol	NA
	17ahprgstrn	NA
	C01953	Pregnenolone
	C02140	Corticosterone
	tststeroneglc	NA
	C00033	Acetic acid
	C04373	Etiocholanolone
	C05138	17a-hydroxypregnenolone
	C05139	16a-Hydroxydehydroisoandrosterone
	C04042	20a-dihydroprogesterone
	C00523	Androsterone
	C00735	Cortisol
	C05284	NA
	C13550	24-hydroxycholesterol
3-oxo-10R-octadecatrienoate beta-oxidation	CE5306	NA
	CE5321	NA
	CE5305	NA
	CE5324	NA
	CE5325	NA
	CE5326	NA
	CE5327	NA
	CE5328	NA
	CE5308	NA
	CE5309	NA
	CE5320	NA
	CE5307	NA
	CE5323	NA
	CE5315	NA
	CE5314	NA
	CE5317	NA
	CE5316	NA
	CE5311	NA
	CE5313	NA
	CE5312	NA

	CE5319	NA
	CE5318	NA
Biopterin metabolism	CE6511	NA
	C00272	Tetrahydrobiopterin
	C03684	Dyspropterin
	C04874	7,8-dihydroneopterin
	CE2705	NA
	C00079	L-phenylalanine
	thbpt4acam	NA
	C04244	6-lactoyltetrahydropterin
	CE5236	NA
	C00268	4a-carbinolamine tetrahydrobiopterin
TCA cycle	C00579	Dihydrolipoamide
	C00311	Isocitric acid
	C00158	Citric acid
	C00033	Acetic acid
	C00248	Lipoamide
	C00042	Succinic acid
	C00417	cis-aconitic acid
Pentose and Glucuronate Interconversions	C00532	L-arabitol
	C00259	NA
	C00379	Xylitol
	C00312	L-threo-2-pentulose
	C00310	D-xylulose
	C00309	D-ribulose

Table S3. List of matched metabolites (KEGG ID and common name) associated with significant metabolic pathways (**Table S2**). NA, not applicable (common name of metabolite was not found).

Query mass	KEGG ID	Common metabolite name
199.9883263	C05838	Coumaric acid
199.9883263	C00811	4-hydroxycinnamic acid
199.9883263	C02763	Enol-phenylpyruvate
199.9883263	C00166	Phenylpyruvic acid
241.084199	C00214	Thymidine
241.084199	C00931	Porphobilinogen
340.1817305	C00219	Arachidonic acid
244.1627814	CE5869	NA
189.052478	C01152	1-methylhistidine
189.052478	C00439	Formiminoglutamic acid

189.052478	C05829	Carglumic acid
269.1152335	CE2615	NA
303.1802775	CE5869	5-aminopentanal
116.071166	C02912	Propylene glycol
116.071166	C00583	NA
116.071166	C00719	Betaine
116.071166	C02917	(S)-propane-1,2-diol
116.071166	C00431	5-aminopentanoic acid
116.071166	C00183	L-valine
106.0046888	C16637	5-deoxyribose-1-phosphate
106.0046888	C00672	Deoxyribose 1-phosphate
106.0046888	C00673	Deoxyribose 5-phosphate
100.0763524	CE1936	NA
158.1262219	C01601	Pelargonic acid
368.1454535	tststerones	NA
115.0758788	C02373	3a,7b,12a-trihydroxy-5a-Cholanoic acid
233.1025392	C08261	Azelaic acid
235.094306	C01239	N-acetyl-b-glucosaminylamine
267.1741585	andrstndn	NA
267.1741585	C00280	Androstenedione
359.2215441	CE2961	NA
359.2215441	C00777	All-trans-retinoic acid
359.2215441	C15493	9-cis-retinoic acid
359.2215441	C00410	Progesterone
191.0707274	C02325	Sinapyl alcohol
325.2008457	C04785	13(S)-HPOT
325.2008457	CE5528	NA
325.2008457	C16321	9(S)-HPOT
317.1149574	CE5309	NA
317.1149574	CE5321	NA
283.2633515	C01530	Stearic acid
378.1996472	C02934	3-ketosphinganine
378.1996472	C00319	Sphingosine
386.1681096	CE7114	NA
386.1681096	CE2053	NA
386.1681096	CE5138	NA
386.1681096	CE5661	NA
386.1681096	CE7115	NA
386.1681096	CE7113	NA
386.1681096	CE7112	NA
386.1681096	CE7088	NA

346.2225912	C02838	L-octanoylcarnitine
101.0240057	C02045	L-erythrulose
101.0240057	C00232	Succinic acid semialdehyde
101.0240057	C06002	(S)-methylmalonic acid semialdehyde
101.0240057	C00349	2-methyl-3-oxopropanoic acid
101.0240057	C00109	3-methyl pyruvic acid
101.0240057	C00164	Acetoacetic acid
217.1075446	CE2576	NA
445.2912285	CE4753	NA
243.1232837	CE2306	NA
243.1232837	CE2305	NA
243.1232837	CE2304	NA
243.1232837	CE2303	NA
133.0498951	C05999	NA
133.0498951	C00246	Butyric acid
133.0498951	C00937	D-lactaldehyde
133.0498951	C00424	Lactaldehyde
133.0498951	C05235	Hydroxyacetone
133.0498951	C00163	Propionic acid
237.1854416	C00750	Spermine
187.0283729	C05842	N1-methyl-2-pyridone-5-carboxamide
187.0283729	C05843	N1-methyl-4-pyridone-3-carboxamide
227.0684328	C00526	Deoxyuridine
277.1800435	C16659	2-ethylidene-1,5-dimethyl-3,3-diphenylpyrrolidine
195.138317	C06078	Polylimonene
195.138317	C15517	Hyodeoxycholic acid
195.138317	C00521	(+)-Limonene
195.138317	C06099	D-limonene
195.138317	C04483	Deoxycholic acid
195.138317	C02528	Chenodeoxycholic acid
195.138317	C06306	(+)-a-pinene
253.1606563	C00951	Estradiol
343.1881661	C16548	N-didesmethyl-tamoxifen
327.2171587	C04717	13-L-hydroperoxylinoleic acid
327.2171587	C14827	9(S)-HPODE
295.22702	C14762	13S-hydroxyoctadecadienoic acid
295.22702	C01595	Linoleic acid
295.22702	C14826	12,13-EpOME
295.22702	C14825	9,10-epoxyoctadecenoic acid

175.0733901	C00147	Adenine
346.2589118	dmnoncrn	NA
183.0655358	C05594	Vanylglycol
257.102469	C00670	Glycerophosphocholine
257.102469	CE0469	NA
271.1183235	CE1661	NA
485.2142769	CE5867	NA
173.0096053	C00158	Citric acid
173.0096053	C04575	Diketogulonic acid
173.0096053	C05422	L-dehydroascorbic acid
173.0096053	C00632	3-hydroxyanthranilic acid
173.0096053	C00311	Isocitric acid
173.0096053	C00425	NA
173.0096053	C00417	cis-aconitic acid
338.187456	C00909	Leukotriene A4
261.1100213	C01074	N-acetylgalactosamine
261.1100213	C01132	NA
261.1100213	C00645	N-acetylmannosamine
261.1100213	C00140	N-acetyl-D-glucosamine
194.9915521	C01041	Ascorbic acid
163.039235	C05583	3-methoxy-4-hydroxyphenylglycolaldehyde
163.039235	C05582	Homovanillic acid
140.9493612	C00086	Urea
349.1495403	C04555	Dehydroepiandrosterone sulfate
152.1154813	C02452	Perillyl alcohol
152.1154813	C11409	(+)-trans-Carveol
152.1154813	C02759	Alpha-pinene-oxide
152.1154813	C00964	(-)-trans-carveol
418.2298909	C01120	Sphinganine 1-phosphate
340.1970345	arachd	NA
340.1970345	eicostet	NA
128.9512245	C06755	Chloroacetic acid
128.9512245	C14860	2,2-dichloro-1,1-ethanediol
175.1334311	C00756	Octanol
263.1256292	CE1926	NA
386.1865804	C05476	Tetrahydrocorticosterone
386.1865804	CE6248	NA
386.1865804	CE6247	NA
386.1865804	CE2569	NA
386.1865804	CE2568	NA
386.1865804	CE5662	NA

386.1865804	CE7092	NA
376.19657	CE0932	NA
376.19657	prist	NA
376.19657	CE2416	NA
506.3174958	C05850	Vitamin K1
193.0500192	CE4970	NA
193.0500192	CE4968	NA
193.0500192	C14784	1,2-dihydroxy-3,4-epoxy-1,2,3,4-tetrahydronaphthalene

Table S4. Matched top 50 metabolites (Query mass, KEGG ID and common name) shown in the heatmap in **Figure 2D**. Metabolites in green are common with those found in biomarker analysis (**Table S5** and **Figure 2F**). NA, not applicable (common name of metabolite was not found for KEGG ID or query mass).

Query mass	KEGG ID	Common metabolite name	Rank freq.	Importance	pCR	RD
167.1433071	NA	NA	0.38	4.61E-07	Low	High
241.084199	C00214	Thymidine	0.26	4.32E-07	High	Low
	C00931	Porphobilinogen	0.26	4.32E-07	High	Low
339.1783589	NA	NA	0.22	4.68E-07	Low	High
244.1627814	CE5869	NA	0.2	4.34E-07	Low	High
189.052478	C01152	1-methylhistidine	0.18	3.77E-07	High	Low
	C00439	Formimino glutamic acid	0.18	3.77E-07	High	Low
	C05829	Carglumic acid	0.18	3.77E-07	High	Low
192.1465677	NA	NA	0.18	3.70E-07	Low	High
537.3270654	NA	NA	0.16	4.21E-07	Low	High
201.0761234	NA	NA	0.16	4.12E-07	High	Low
184.9697152	NA	NA	0.16	4.01E-07	Low	High
116.1074193	NA	NA	0.16	3.28E-07	Low	High
455.1891668	NA	NA	0.14	4.05E-07	Low	High
233.1025392	C08261	Azelaic acid	0.14	3.75E-07	High	Low
317.1965147	NA	NA	0.12	4.50E-07	Low	High
340.2486084	NA	NA	0.12	4.08E-07	High	Low
265.1410739	NA	NA	0.12	4.02E-07	Low	High

Table S5. Most important metabolites (Query mass, KEGG ID, and common ID) of a selected model ranked from most to least important (analyzed using biomarker analysis; **Figure 1E**). NA, not applicable (common

name of metabolite was not found for KEGG ID or Query mass). Metabolites in green are common with those found in GSEA analysis (**Table S4; Figure 1 F**).

Metabolic pathway	Total compound	Hits	Raw *P*-value	FDR
Ketone body metabolism	13	1	0.028041	0.22847
Butyrate metabolism	19	1	0.028041	0.22847
Mitochondrial electron transport chain	19	1	0.028041	0.22847
Phytanic acid peroxisomal oxidation	26	1	0.028041	0.22847
Warburg effect	58	3	0.02965	0.22847
Pyruvate metabolism	48	3	0.032217	0.22847
Gluconeogenesis	35	2	0.034768	0.22847
Fatty acid biosynthesis	35	4	0.09858	0.33608
Oxidation of branched-chain fatty acids	26	3	0.098948	0.33608
Carnitine synthesis	22	2	0.10051	0.33608
Estrone metabolism	24	3	0.10613	0.33608
Citric acid cycle	32	2	0.10907	0.33608
Glutamate metabolism	49	2	0.10907	0.33608
Beta-alanine metabolism	34	1	0.12236	0.33608
Arginine and proline metabolism	53	2	0.12405	0.33608
D-arginine and D-ornithine metabolism	11	1	0.1242	0.33608
Urea cycle	29	1	0.1242	0.33608
Mitochondrial beta-oxidation of short-chain saturated fatty acids	27	2	0.16536	0.34363
Methionine metabolism	43	2	0.1821	0.34363
Phospholipid biosynthesis	29	1	0.19058	0.34363
Betaine metabolism	21	1	0.19058	0.34363
Phosphatidylcholine biosynthesis	14	1	0.19058	0.34363
Phosphatidylethanolamine biosynthesis	12	1	0.19058	0.34363
Porphyrin metabolism	40	1	0.19269	0.34363
Valine, leucine and isoleucine degradation	60	4	0.22169	0.34363
Propanoate metabolism	42	2	0.22171	0.34363
Ammonia recycling	32	1	0.24296	0.34363
Alanine metabolism	17	1	0.24296	0.34363
Biotin metabolism	8	1	0.24296	0.34363

Threonine and 2-oxobutanoate degradation	20	1	0.24296	0.34363
Transfer of acetyl groups into mitochondria	22	1	0.24296	0.34363
Histidine metabolism	43	1	0.2681	0.34363
Sulfate/sulfite metabolism	22	1	0.26889	0.34363
Androgen and estrogen metabolism	33	1	0.26889	0.34363
Tyrosine metabolism	72	1	0.27015	0.34363
Thyroid hormone synthesis	13	1	0.27015	0.34363
Pantothenate and CoA biosynthesis	21	2	0.2764	0.34363
Phenylacetate metabolism	9	1	0.30003	0.3632
Bile acid biosynthesis	65	1	0.34098	0.40218
Steroidogenesis	43	1	0.35236	0.40521
Steroid biosynthesis	48	1	0.3811	0.42757
Ubiquinone biosynthesis	20	1	0.39437	0.43193
Vitamin K metabolism	14	1	0.45489	0.48662
Fatty acid metabolism	43	1	0.49327	0.49327
Beta-oxidation of very-long-chain fatty acids	17	1	0.49327	0.49327
Mitochondrial beta-oxidation of long-chain saturated fatty acids	28	1	0.49327	0.49327

Table S6. Metabolic pathways differentially regulated in RD vs. pCR patients with BC (identified using MSEA; **Figure 3A, B**). Pathways in red have $p \leq 0.05$. Pathways highlighted in green are common metabolic pathways obtained through MSEA and GSEA. Total compound indicates the total number of compounds in a particular pathway. Hits indicate the matched number of compounds from user-uploaded data. FDR, false discovery rate.

Supplementary Figures

Figure S1

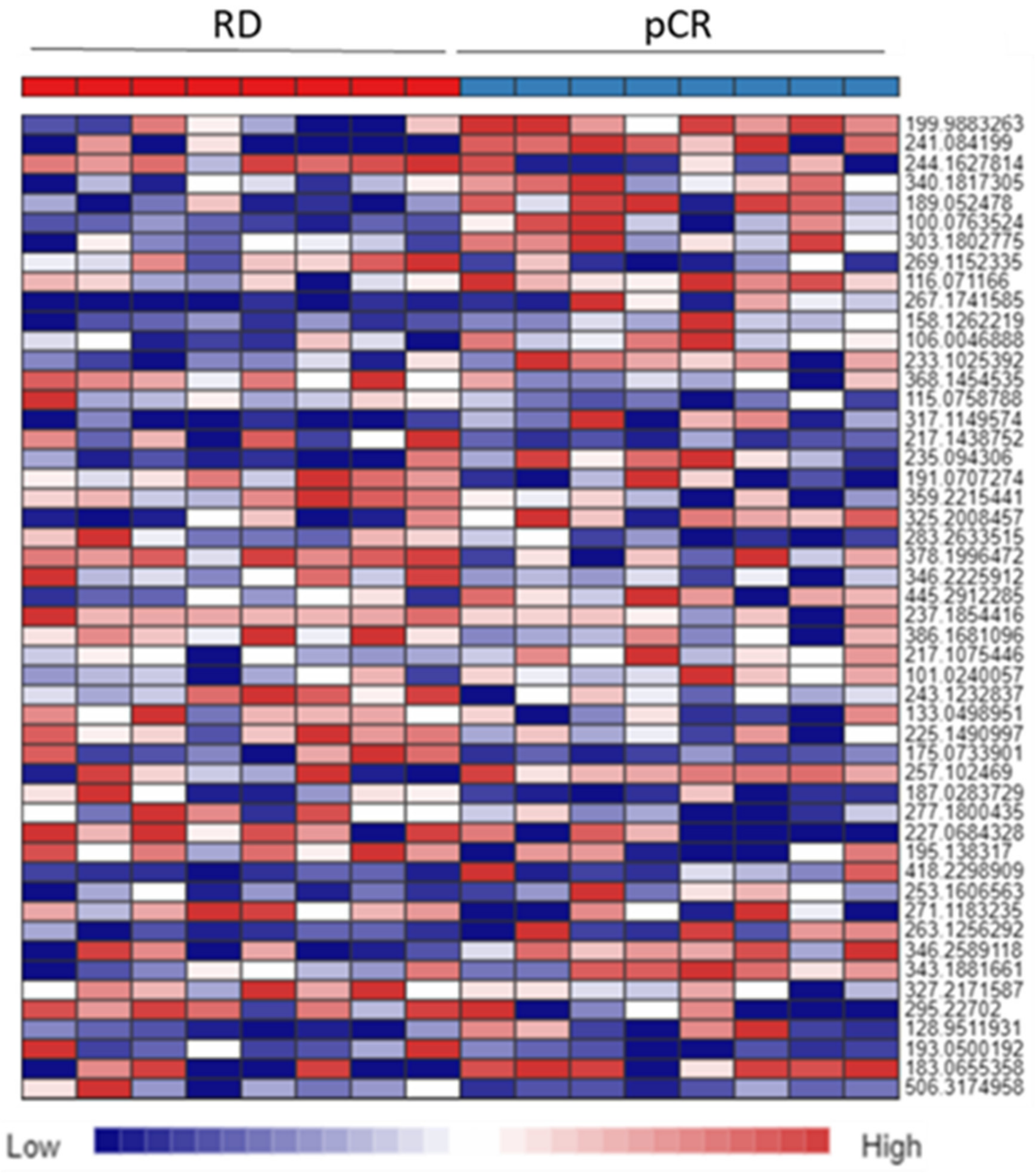

Figure S1. Heatmap showing the top 50 differentially predicted metabolites in RD vs. pCR patients identified using GSEA.

Figure S2

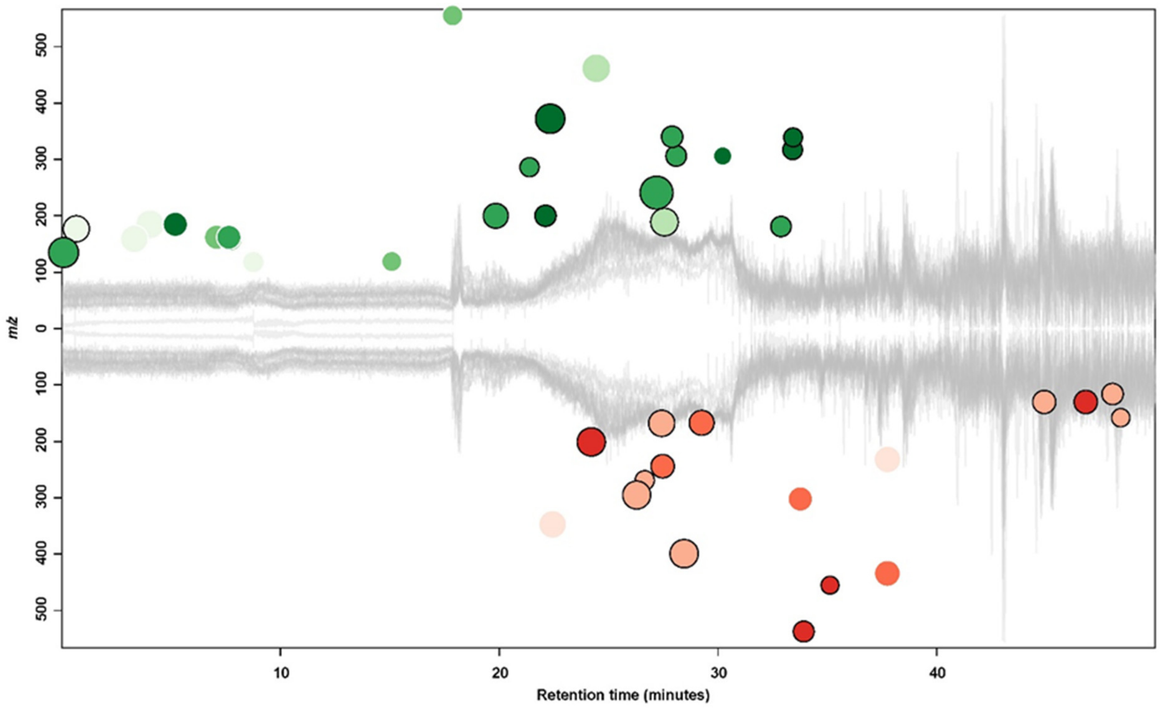

Figure S2. Cloud plot showing significantly altered metabolites in exosomes from patients with pCR. Features with increased intensity in exosomes from patients with pCR are shown in green, whereas features with decreased intensity in exosomes from patients with pCR are shown in red. The size of each bubble corresponds to the log-fold change of the feature. Features with p value ≤ 0.01 and fold change > 1.5 are represented in the cloud plot.

Figure S3

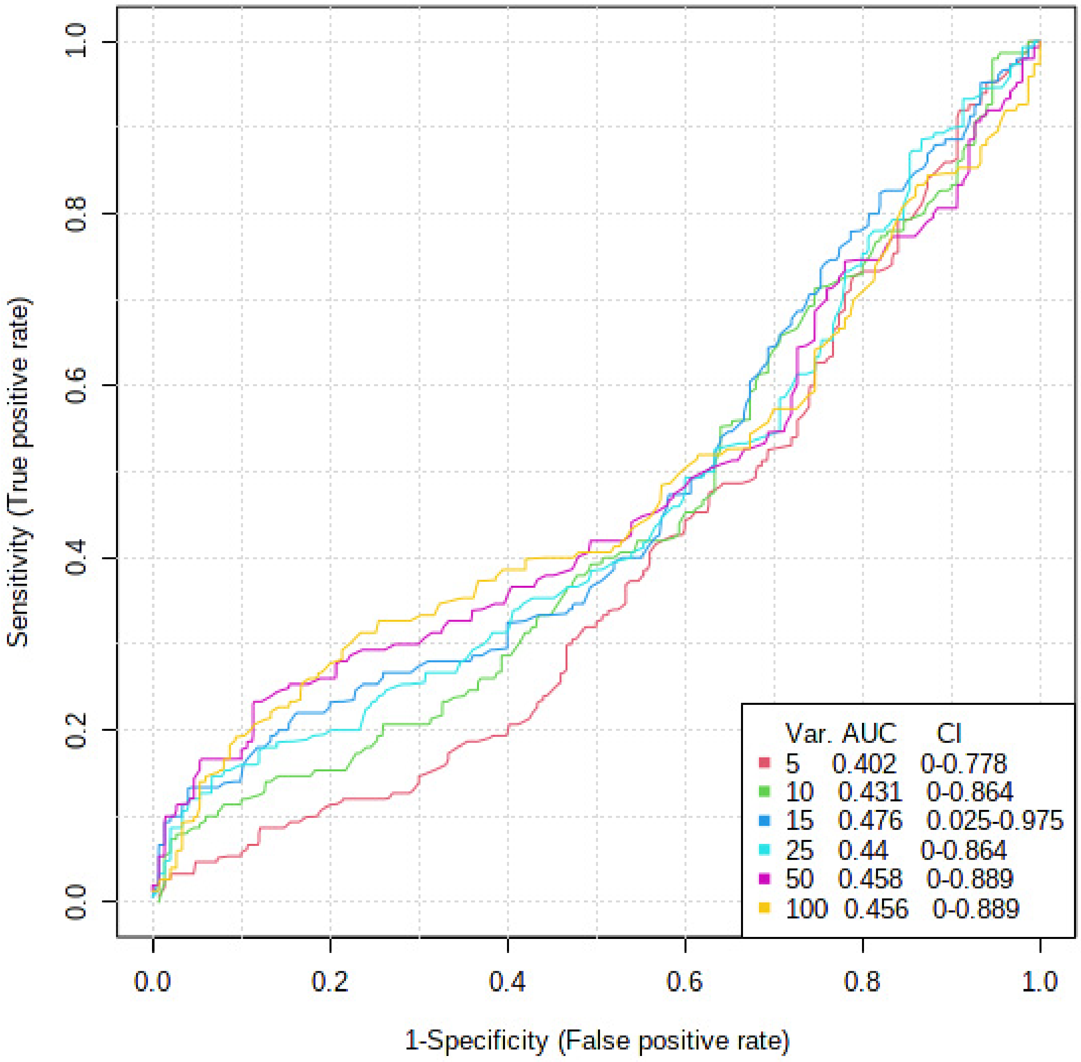

Figure S3. Plot of ROC curves for all or a single biomarker model based on its average performance across all MCCV runs. For a single biomarker, the 95 percent confidence interval can be computed and will appear as a band around the ROC curve.
